# Effects of Static or Oscillating Dietary Crude Protein Levels on Fermentation Dynamics of Beef Cattle Diets Using a Dual-Flow Continuous Culture System

**DOI:** 10.1371/journal.pone.0169170

**Published:** 2016-12-30

**Authors:** Paloma de Melo Amaral, Lays Débora Silva Mariz, Pedro Del Bianco Benedeti, Lorrayny Galoro da Silva, Eduardo Marostegan de Paula, Hugo Fernando Monteiro, Teshome Shenkoru, Stefanie Alvarenga Santos, Simon Roger Poulson, Antonio Pinheiro Faciola

**Affiliations:** 1 Department of Agriculture, Nutrition, and Veterinary Sciences, University of Nevada, Reno, Nevada, United States of America; 2 Department of Animal Sciences, Federal University of Viçosa, Viçosa, Minas Gerais, Brazil; 3 Department of Animal Sciences, Maringá State University, Maringá, Paraná, Brazil; 4 Department of Animal Sciences, Federal University of Bahia, Salvador, Bahia, Brazil; 5 Department of Geological Sciences & Engineering, University of Nevada, Reno, Nevada, United States of America; National Institute for Agronomic Research, FRANCE

## Abstract

The objective of this study was to evaluate the effects of increasing dietary crude protein (CP) levels and also comparing the effects of static versus oscillating dietary CP on ruminal nutrient digestibility, ruminal fermentation, nitrogen (N) metabolism, and microbial efficiency in beef cattle diets using a dual-flow continuous culture system. Eight fermenters (1,223 ± 21 mL) were used in a replicated 4 x 4 Latin square design with periods lasting 12 d each (8 d for adaptation and 4 d for sampling). Dietary treatments were: 1) 10% CP, 2) 12% CP, 3) 14% CP, and 4) 10 and 14% CP diets oscillating at 48-h intervals. Experimental diets consisted of 50% orchard hay and 50% concentrate. Fermenters were fed 72 g/d and solid and liquid dilution rates were adjusted to 5.5 and 11%/h, respectively. Data were analyzed using the MIXED procedure in SAS with α = 0.05. Apparent and true ruminal digestibilities of dry matter and organic matter were not affected (P > 0.05) by increasing dietary CP, nor by oscillating dietary CP. Total volatile fatty acids concentration and molar proportions of acetate, propionate, butyrate, valerate, iso-butyrate and iso-valerate were not affected (P > 0.05) by increasing or oscillating dietary CP. Ruminal NH_3_-N concentration increased linearly (*P* < 0.01) in response to increasing dietary CP. Total N, non-ammonia N, and rumen undegraded protein flows did not differ among treatments or between oscillating dietary CP and static 12% CP. Microbial N and NH_3_-N flows and microbial efficiency did not differ when comparing oscillating versus static CP (P > 0.05). However, there was a quadratic effect (*P* < 0.05) for these variables when dietary CP was increased. These results indicate that either ruminal microorganisms do not respond to oscillating CP levels or are capable of coping with 48-h periods of undernourishment.

## Introduction

In general, ruminant animals convert about 20–30% of their dietary nitrogen (**N**) into animal protein and about 70–80% is excreted in the urine and feces [1] [2] [3]. Positive responses in animal performance associated to increasing dietary N [4] [5] coupled to the risk of losing production has led to excessive N feeding over the years [6] [7] [8], which has economic and environmental implications. Protein is a costly nutrient; moreover, excessive dietary N is harmful to the environment because excreted N accumulates in the atmosphere, soil, and groundwater [9]. Therefore, efforts to improve N utilization in ruminants are of utmost importance.

In nature several animal, microorganism, and plant species experience seasonal periods of undernourishment followed by periods of nutrient abundance. This nutrient oscillation seems to affect the homeostatic and homeorhetic processes in a manner that promotes a period of accelerated growth previously defined as compensatory growth [10] [11] [12]. Studies have demonstrated that feeding diets with oscillating crude protein (**CP**) levels can enhance N retention in growing sheep [13] [14] [1] and finishing cattle [4] [5]. However, the mechanisms associated with this enhanced N utilization have been mostly studied from the host animal standpoint (for example, recycling N via saliva as studied by Doranalli et al. [1], or by increased dry matter intake as observed by Ludden et al. [15] but not from the perspective of the ruminal microbial population. It may be possible that the ruminal microorganisms increase their efficiency of N utilization when N supply oscillates below and above their requirements [4]. Thus animals fed oscillating levels of CP would have a greater flow of microbial protein when compared to animals fed constant levels of CP.

The objective of this study was to evaluate the effects of increasing dietary CP levels and compare the effects of static versus oscillating dietary CP on nutrient digestibility, ruminal fermentation, ruminal N metabolism, and microbial efficiency in beef cattle diets, using a dual-flow continuous culture system. We hypothesized that feeding oscillating dietary CP would enhance ruminal N metabolism and microbial efficiency in a dual-flow continuous culture system.

## Material and Methods

### Ethical Approval

All animal care and handling were approved by the University of Nevada, Reno Institutional Animal Care and Use Committee (IACUC, protocol # 00588). Ruminal cannulations were conducted as described in Benedeti et al. [16].

### Experiment Design and Diets

Eight 1,223 ± 21 mL dual-flow continuous culture fermenters, similar to that described by Hoover et al. [17], were used in four consecutive periods lasting 12 d each (8 d for diet adaptation and 4 d for sample collection). The study was conducted as a replicated 4 x 4 Latin square design with four treatments and four periods, totaling eight replicates per treatment.

Dietary treatments consisted of: 1) static 10% CP diet, 2) static 12% CP diet, 3) static 14% CP diet, and 4) oscillating 10 and 14% CP diets every 48-h (**OSC**). Experimental diets were composed of 50% orchard hay and 50% concentrate [dry matter (**DM**) basis] and were formulated based on the requirements outlined in the beef NRC [18]. Dietary ingredients were ground to pass a 2 mm sieve using a Wiley mill (Model #2, Arthur H. Thomas Co., Philadelphia, PA) and analyzed for their chemical composition. Ingredient and chemical composition of experimental diets are shown in Table 1.

**Table 1:** Ingredient and chemical composition of experimental diets.

Item^1^	Treatment, CP%		
	10%	12%	14%
Ingredient, % DM			
Orchard Hay	50.0	50.0	50.0
Dry, ground corn	46.9	42.5	38.1
Solvent extracted soybean meal	2.1	6.5	10.9
Mineralized salt^2^	1.0	1.0	1.0
Composition			
DM, %	88.8	89.2	89.6
OM, % of DM	94.6	94.3	94.0
NDF, % of DM	38.3	38.2	38.1
CP, % of DM	10.0	12.0	14.0
EE, % of DM	2.3	2.4	2.5
ME^3^, Mcal/kg	2.8	2.8	2.7

^1^DM = dry matter; OM = organic matter; NDF = neutral detergent fiber; CP = crude protein; EE = ether extract; ME = metabolizable energy.

^2^Provided (per kg of DM): 100 g of sodium chloride, 12.5 g of zinc, 12.5 g of iron, 12.5 g of manganese, 1,750 ppm of copper, 450 ppm of iodine, and 240 ppm of cobalt.

^3^Metabolizable energy was calculated according to the beef NRC [18].

### Dual-flow Continuous Culture System Operation and Sample Collection

Ruminal fluid was collected from the ventral, central, and dorsal regions of the rumen two hours after morning feeding from two ruminally cannulated Aberdeen Angus steers (average body weight of 785 kg) fed a diet composed of 50% orchard hay and 50% concentrate. The rumen fluid was immediately strained through four layers of cheesecloth, and approximately 10 L of ruminal fluid were collected and transported to the laboratory in a pre-warmed thermos flask (39 ±0.5°C). The rumen fluid was homogenized, infused with N_2_ to maintain the anaerobic environment and adjusted to 39°C by submerging a 5,000 mL Erlenmeyer flask in a pre-heated water bath.

**Fig 1:**
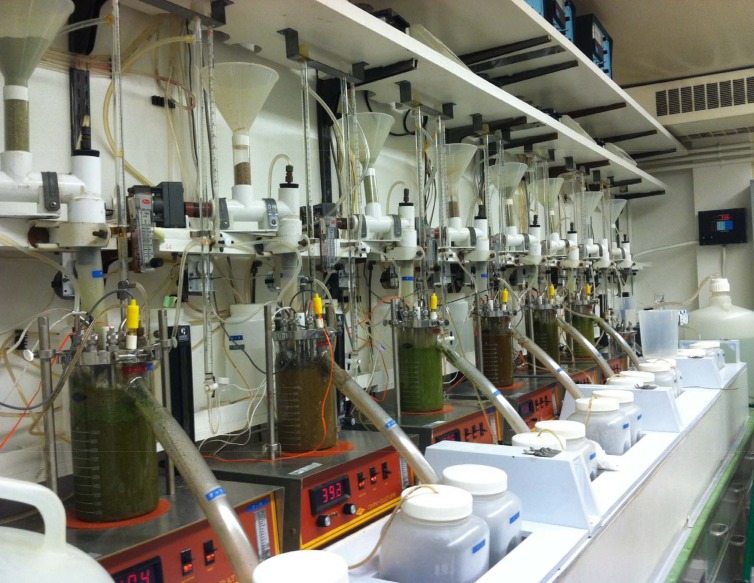
Dual-flow continuous culture system right view.

**Fig 2:**
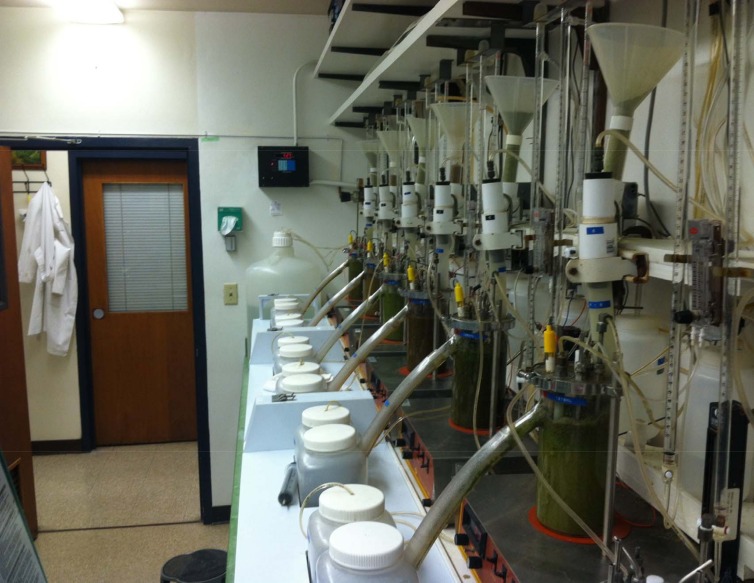
Dual-flow continuous culture system left view.

**Fig 3:**
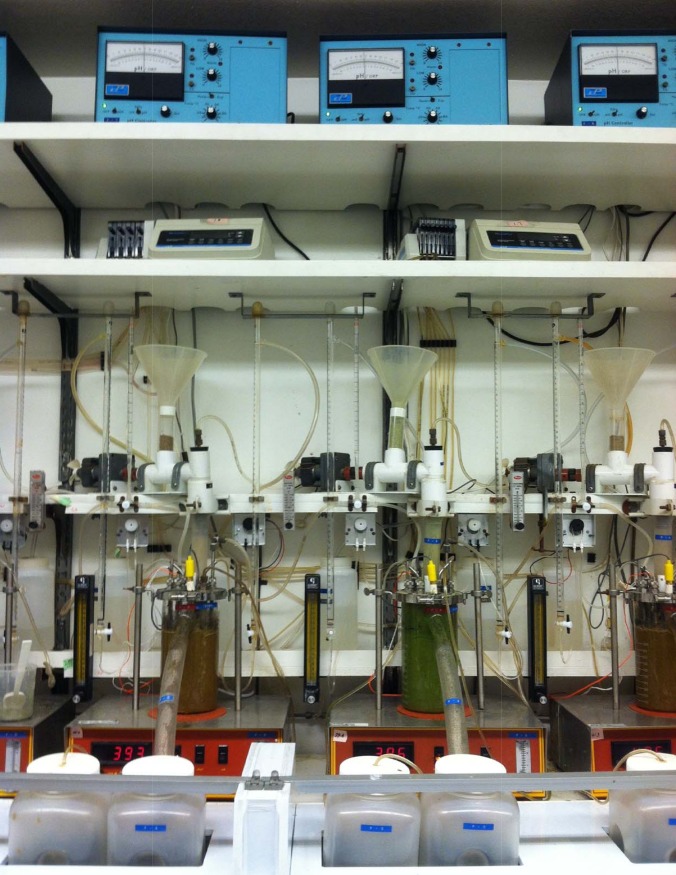
Dual-flow continuous culture system frontal view.

About 1,250 mL of liquid was then poured into each of the fermentation jars until it cleared the overflow spout (Figs 1–3). The temperature was maintained at 39°C and N_2_ (40 mL/min) was infused into the fermenters to maintain the anaerobic conditions of the system, as described by Hoover et al. [17]. However, pH was not controlled and urea was added to the artificial saliva [19] to simulate recycled N. The pH of each fermenter was monitored using individual Cole-Parmer pH controllers (Model 5997–20). A central propeller apparatus driven by magnets was used to stir continuously the fermenters contents at the rate of 150 rpm. Solid and liquid dilution rates were adjusted to 5.5 and 11%/h, respectively, by adjusting the artificial saliva infusion and filtered liquid flows. Saliva was continuously infused at 2.1 mL/min and measured twice daily for consistency.

Fermenters were fed a total of 72 g DM/d divided in two equal portions at 0900 and 2100 h. Pelleted orchard hay and concentrate were combined in each feeding time.

Digesta effluent (solid and liquid separated) were collected in 4 L plastic containers. At 0830 h of each d of the adaptation period, the containers were weighed and contents were discarded. On days 8 to 12, each container received 20 mL of 50% H_2_SO_4_ and were maintained submerged (approximately two-thirds of their depth) in a water bath at 4°C to prevent microbial and enzymatic activities.

On days 9, 10, 11 and 12 of each period, liquid and solid overflow output from each fermenter were combined and homogenized using a mixer (T25 basics, IKA Works, Inc., Wilmington, NC). Then, a 500 mL sample was collected via vacuum system and stored at -20°C. The 500 mL overflow samples collected on each of the 4 collection days were composited by fermenter per period. The overflow composite (2,000 mL/fermenter per period) was mixed, and a 300 mL subsample was collected, freeze-dried, and ground using pestle and mortar. The samples were placed in a plastic container for further chemical analyses (detailed below). An additional overflow sample was filtered through two layers of cheesecloth and two 10 mL aliquots of fluid were preserved with 0.2 mL of a 50% H_2_SO_4_ solution for later determination of ammonium nitrogen (**NH**_**3**_**-N**) and volatile fatty acids (**VFA**) as described below.

On day 7, before beginning the infusion of ^15^N, an effluent sample (solid and liquid) of each fermenter was collected, to determine the background ^15^N abundance. Then, 0.077 g of ammonium sulfate enriched with 10 atom% ^15^N [ammonium sulfate ^15^(NH_4_)_2_SO_4_] (Sigma-Aldrich Co., St. Louis, MO) was infused into each fermenter to instantaneously label the NH_3_-N pool. Saliva was reformulated and 0.077 g/L of enriched ^15^(NH_4_)_2_SO_4_ (Sigma-Aldrich Co., St. Louis, MO) were added in replacement of isonitrogenous amounts of urea to maintain a steady-state concentration of ^15^N enrichment in fermenters.

On days 10 and 12, the ruminal pH of each fermenter was measured at 0, 1, 2, 4, 6, 8, and 10 h after feeding, using an Accumet portable AP61 pH meter (Fisher Scientific, Atlanta, GA).

On the last day of each period, the entire fermenter content was strained through two layers of cheesecloth and the liquid phase was centrifuged at 1,000 g for 10 min, at 4°C (Sorvall RC-5B Refrigerated Super speed Centrifuge, DuPont Instruments, Wilmington, DE) to remove feed particles. Then, the supernatant was centrifuged at 10,000 g for 20 min [20]. Supernatant was discarded and bacterial pellets were freeze dried and stored for further analysis.

### Chemical Analyses and Calculations

Feed and digesta effluent samples were analyzed for DM (method 934.01), ash (method 938.08), crude protein (CP; method 984.13), and ether extract (method 920.85) according to AOAC [21]. The organic matter (**OM**) was calculated as the difference between DM and ash contents. For neutral detergent fiber (**NDF**) and acid detergent fiber (**ADF**), samples were sequentially analyzed, treated with alpha thermo-stable amylase without sodium sulfite according to Van Soest et al. [22] and adapted for the Ankom200 Fiber Analyzer (Ankom Technology, Macedon, NY). Samples of microbial pellet and digesta effluent background were analyzed for DM, CP, and ash as detailed previously for feed samples.

Volatile fatty acid concentrations in the digesta effluent were determined using gas chromatography (Varian Model 3800; Varian, Inc, Walnut Creek, CA; equipped with a glass column [180 cm x 4 mm i.d.]) packed with GP 10% SP-1200/1% H_3_PO_4_ on 80/100 Chromosorb WAW [Supelco, Bellefonte, PA]), and N was used as a carrier gas at a flow rate of 85 mL/min^-1^. The NH_3_-N concentrations (on fermenter and effluent digesta) were determined by colorimetric as described by Chaney and Marbach [23].

Background, digesta effluent and microbial pellets samples were analyzed for total N and ^15^N enrichment according to Werner et al. [24]. Isotope analyses were performed using an Eurovector model 3028 elemental analyzer interfaced to a Micromass Isoprime stable isotope ratio mass spectrometer. Microbial N flow and microbial efficiency were calculated as follows: Microbial N flow (expressed in g/d) = [non-ammonia N (**NAN**) flow * percentage of ^15^N atom excess of digesta effluent]/(percentage of ^15^N atom of microbial pellet), with ^15^N digesta effluent background subtracted from ^15^N enrichment. Microbial efficiency = Microbial N flow (g) / OM truly digestible (kg) [25]. According to Soder et al. [26] and Benedeti et al. [16] digestibilities were calculated as follows (using DM as an example): DM apparently digested (%) = [(g of DM intake—g of effluent flow DM) / g of DM intake] * 100; DM truly digested (%) = {[g of DM intake—(g of effluent flow DM—g of microbial DM)] / g of DM intake} * 100. Effluent was corrected for grams of buffer in both equations.

### Statistical Analysis

Statistical analysis was conducted in a replicated 4 x 4 Latin square design using the MIXED procedures in SAS software (version 9.2), according to the following model:

Yijk=μ+Ti+fj+pk+eijk

where *Y*_*ijk*_ = dependent variable measured in fermenter *j* that was subjected to the *i* treatment in period *k*; *μ* = general mean, *Ti* = fixed effect of treatment *i*, *fj* = random effect of fermenter *j*, *pk* = random effect of period *k*, and *eijk* = random error assuming NID (0; σ^2^_ε_).

In this analysis, the fixed effect was represented by protein levels and the random effects were represented by fermenter and period effects. The fixed effects for evaluating pH in the 4 x 4 Latin square were protein levels (P), collection time (T), and the interaction between these two factors (P * T). A scheme of repeated in time measurements was used [27], with collection times (0, 1, 2, 4, 6, 8 and 10 h after feeding) repeated once within each experimental unit (fermenter * period).

Protein level comparisons followed the decomposition of orthogonal polynomials in linear and quadratic effects to compare 10, 12, and 14% CP levels and were conducted using the MIXED procedures in SAS software (version 9.2). Moreover, an orthogonal contrast was made to compare oscillating dietary CP *vs*. 12% static CP treatments. Homogeneity of variances between treatments was assumed and the degrees of freedom were estimated by using the Kenward-Roger method. All statistical procedures were conducted using 0.05 as the critical probability level for a type I error.

## Results

### Apparent and True Ruminal Digestibility

Apparent ruminal digestibilities of DM, OM, NDF, and ADF were not affected (*P* > 0.05) by increasing dietary CP, nor by oscillating dietary CP compared with 12% static CP. Similarly, there were no differences (*P* > 0.05) in true ruminal digestibility of DM and OM among treatments (Table 2).

**Table 2:** Effects of dietary CP levels or oscillating dietary CP on apparent and true ruminal digestibilities of dietary nutrients in dual-flow continuous culture system.

Item^1^	Treatment, CP%				SEM	*P*-value		
	10%	12%	14%	OSC		OSC vs. 12%	Linear	Quadratic
Apparent Ruminal Digestibility, %								
DM	21.0	22.4	19.9	21.2	1.52	0.55	0.59	0.27
OM	24.1	24.6	20.9	22.9	1.44	0.30	0.07	0.16
NDF	36.3	38.9	37.7	39.7	3.40	0.82	0.72	0.59
ADF	21.3	20.4	20.8	23.9	2.77	0.30	0.87	0.83
True Ruminal Digestibility, %								
DM	41.8	39.0	39.1	43.6	2.21	0.15	0.39	0.61
OM	39.2	37.2	36.0	39.0	1.88	0.46	0.21	0.84

^1^DM = dry matter; OM = organic matter; NDF = neutral detergent fiber; ADF = acid detergent fiber.

### Volatile Fatty Acids and Ruminal pH

**Fig 4:**
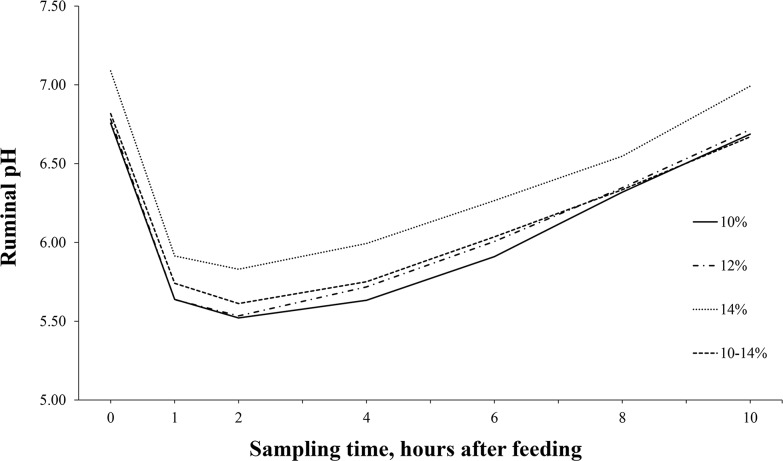
Effects of dietary CP levels or oscillating dietary CP on ruminal pH at different times after feeding in dual-flow continuous culture system.

There was a significant positive linear effect (*P* < 0.01) on ruminal pH with increasing dietary CP levels (Table 3). However, no significant difference in ruminal pH was observed (*P*>0.05) between oscillating dietary CP and 12% static CP. Interactions between diets and time were not significant (P>0.05). Fig 4 shows ruminal pH pattern at different times after feeding, ruminal pH was greater with the 14% CP level. Total VFA concentration and individual VFA molar proportions (acetate, propionate, butyrate, valerate, iso-butyrate and iso-valerate) were not affected (P > 0.05) by dietary CP levels or oscillating dietary CP.

**Table 3:** Effects of dietary CP levels or oscillating dietary CP on ruminal pH, total VFA concentration and individual VFA proportions in dual-flow continuous culture system.

Item^1^	Treatment, CP%				SEM	*P*-value		
	10%	12%	14%	OSC		12% vs. OSC	Linear	Quadratic
pH	6.1	6.1	6.4	6.1	0.08	0.70	<0.01	0.12
Total VFA, m*M*	60.5	64.7	64.7	63.0	3.37	0.58	0.17	0.41
VFA, % total								
Acetate	35.6	34.5	30.9	38.8	2.99	0.29	0.25	0.72
Propionate	28.9	31.2	33.2	28.9	2.38	0.41	0.14	0.95
Butyrate	29.1	29.1	30.5	26.1	3.37	0.51	0.76	0.85
Valerate	2.7	2.4	2.7	2.6	0.39	0.58	0.99	0.47
*Iso-*Butyrate	0.1	0.4	0.5	0.5	0.51	0.23	0.70	0.36
*Iso-*Valerate	3.1	2.5	2.3	3.0	0.54	0.38	0.19	0.64
BCVFA, m*M*	2.2	1.9	1.8	2.2	0.40	0.49	0.34	0.75
Acetate: propionate	1.3	1.1	1.0	1.4	0.12	0.07	0.05	0.83

^1^VFA = volatile fatty acids; BCVFA = Branched-chain VFA.

### Nitrogen Metabolism and Microbial Efficiency

There was quadratic effect on ruminal NH_3_-N concentration (*P* < 0.05) in response to increasing dietary CP (Table 4). Digesta effluent ruminal NH_3_-N concentration was about three times greater in the 14% CP treatment compared to the 10 and 12% CP treatments. Flows of total N, NAN, and RUP, and CP digestibility did not differ among treatments.

Flows of microbial N and NH_3_-N, and microbial efficiency did not differ when compared oscillating dietary CP versus static 12% CP (P > 0.05). However, it was observed a quadratic effect (*P* < 0.05) in these variables when dietary CP was increased.

**Table 4:** Effects of dietary CP levels or oscillating dietary CP on ruminal nitrogen metabolism in dual-flow continuous culture system.

Item^1^	Treatment, CP%				SEM	*P*-value		
	10%	12%	14%	OSC		12% vs. OSC	Linear	Quadratic
NH_3_-N, mg/100 mL	2.9	3.1	8.9	4.8	1.10	0.12	<0.01	<0.01
CP digestibility, %	77.5	70.0	67.0	68.4	7.43	0.86	0.30	0.79
Nitrogen flow, g/d								
Total N	1.8	2.0	2.1	1.8	0.15	0.27	0.30	0.81
NH_3_-N	0.1	0.1	0.3	0.1	0.03	0.11	<0.01	<0.01
NAN	1.8	1.9	1.8	1.9	0.09	0.70	0.72	0.24
Microbial N	0.9	1.1	1.0	1.1	0.04	0.78	0.06	0.01
Microbial efficiency^2^	31.3	40.5	37.4	36.5	2.20	0.19	0.05	0.02
RDP supply, g of N/d^3^	0.8	1.1	1.2	1.1	0.07	0.84	<0.01	0.34
RUP flow, g of N/d^4^	0.9	0.9	1.0	0.9	0.07	0.95	0.27	0.34

^1^NH_3_-N = ammonia nitrogen; CP = crude protein; NAN = non-ammonia nitrogen; OM = organic matter.

^2^Microbial efficiency = g of microbial N/kg of DM truly digested.

^3^RDP supply = rumen-degraded protein supply = total N flow–microbial N. [28]

^4^RUP flow = rumen-undegraded protein flow = total N intake–RUP flow. [28]

## Discussion

### Apparent and True Ruminal Digestibility

The results from this experiment showed that increasing dietary CP had no significant effect on *in vitro* DM, OM, and fiber ruminal digestibilities. These observations are in agreement with previous reports in beef cattle [29] [30]. This has also been observed in studies with oscillating dietary CP between 10 and 15% at 48-h intervals [13]. Archibeque et al. [31] reported that steers fed increasing CP levels or oscillating dietary CP levels had greater DM digestibility, ranging from 71.8% (9.1% CP) to 77.7% (14.9% CP), and 77.5% when oscillating from 9.1% to 14.9% dietary CP. Factors such as CP levels and sources may affect the response of CP on digestibility [32] [33].

It was not anticipated that dietary CP would affect DM, OM, and fiber digestibilities since N metabolism and microbial efficiency were the main focus of this study. Diets were formulated to be as similar as possible so potential confounding effects could be minimized and N metabolism and microbial efficiency could be evaluated more carefully.

The values observed in the present study are similar to Bach et al. [20] that tested three protein sources in continuous culture fermenters and reported true OM ruminal digestibility of 44.6%. Fiber digestibilities observed were lower than expected and this was due to low quality forages available at the time. The results of the present study indicate that increasing dietary CP from 10 up to 14% or oscillating dietary CP between 10–14% at 48-h intervals have no effects on ruminal digestibility in beef cattle diets.

### Volatile Fatty Acids and Ruminal pH

Volatile fatty acids are an end product of ruminal fermentation and as expected are influenced by ruminal digestibility. In the present study it was not expected changes in ruminal digestibility of DM, OM, and fiber; therefore, changes in total VFA and individual VFA molar proportion were unlikely to occur. Similarly to our observations, other studies have reported that increasing dietary CP levels had no significant effect on total VFA concentration [29] [33].

Factors that affect total VFA concentration and individual VFA molar proportions include level of intake, ruminal digestibility (DM, OM, and fiber), passage rate, dietary composition, and forage to concentrate ratio [33] [34]. Because these variables did not change in the present study it is sensible and consistent to expect that total VFA concentration and individual VFA molar proportions would not change. The results of the present study indicate that increasing dietary CP from 10 up to 14% or oscillating dietary CP between 10–14% at 48-h intervals have no effects on total VFA concentration and individual VFA molar proportions in beef cattle diets.

In the present study ruminal pH mean varied from 6.1 to 6.4 indicating an adequate ruminal environment for microbial activity [35]. Ruminal pH pattern was typical of a two-time feeding regime, with the minimum pH value observed about 2 h after feeding, which indicates that ruminal fermentation was probably greatest at this time.

### Nitrogen Metabolism and Microbial Efficiency

Our hypothesis was that feeding oscillating dietary CP would enhance ruminal N metabolism and microbial efficiency in a dual-flow continuous culture system. Because compensatory growth has been observed in different animal species [1] [5] the goal of the present study was to assess the ruminal microbial response to oscillating dietary CP independently of other variables in order to minimize any potential confounding effects. In order to achieve so, it was designed an experiment in which diets were as similar as possible (with the exception of dietary CP levels); furthermore, DMI and passage rate were adjusted to be the same among diets, which would eliminate possible DMI effects as observed by Krehbiel et al. [15], which could compromise the interpretation of the results. Also, in the present study, recycling N via saliva was kept constant among treatment, which would eliminate possible interferences as observed by Doranalli et al. [1]. To our knowledge, the present experiment is the first to control these factors in order to evaluate the effects of oscillating dietary CP on ruminal N metabolism and microbial efficiency in the rumen.

As expected, ruminal NH_3_-N linearly increased when dietary CP levels increased and this has been previously reported by Chanthakhoun et al. [36] and Chen et al. [37], because ruminal NH_3_-N is an end product of protein degradation it was expected that increased dietary CP levels would increase ruminal NH_3_-N concentration. Ruminal NH_3_-N concentration of 2.4 mg/100 mL have been reported as the minimum concentration for adequate microbial growth [38] [39], which is within the levels reported in the present study. Interestingly, ruminal NH_3_-N concentration did not differ between static 12% CP and oscillating dietary CP between 10–14% at 48-h intervals. This indicates that ruminal protein degradation and ruminal microbial assimilation of NH_3_-N is not affected by 48-h CP oscillation and this could be due to microbial adaptation within 48-h or maybe microbial capacity to cope during periods of undernourishment. Other oscillation regimes (for example: 24-h high CP followed by 48-h low CP) could help elucidate these issues.

The results of the present study indicate that increasing dietary CP from 10 up to 14% or oscillating dietary CP between 10–14% at 48-h intervals have no effects on ruminal CP digestibility in beef cattle diets.

In the present study it was observed a linear increase in NH_3_-N flow, which is consistent with our expectation and previously reported studies [40] [41]. It was also observed a quadratic increase in microbial N flow, indicating that 12% CP would allow the greater microbial protein growth in the rumen and beyond that level, there would be no further benefit of feeding greater dietary CP. This is in agreement with other studies that reported 12% CP as being optimal for microbial protein growth in the rumen [42] [43].

In the present study it was observed a quadratic increase in microbial efficiency, indicating that 12% CP would allow the greater microbial efficiency in the rumen (calculated as g of microbial N/kg of OM truly digested) and beyond that CP level, there would be no further benefit of feeding greater dietary CP. This is in agreement with other studies that reported 12% CP as being optimal for microbial protein growth in the rumen [42].

The results of the present study indicate that increasing dietary CP from 10 up to 14% or oscillating dietary CP between 10–14% at 48-h intervals have no effects on RUP supply in beef cattle diets and this may be related to the source of CP used (solvent extracted soybean meal), which is highly degradable in the rumen.

In the present study it was observed a linear increase in RDP supply in the fermenter, which is consistent with our expectation and previously reported studies [44]. Because of the CP source used, it was expected that greater dietary CP levels would promote greater RDP supply.

The most important finding of the present study was that contrary to our hypothesis, oscillating dietary CP between 10–14% at 48-h intervals had no effects on ruminal N metabolism and microbial efficiency when compared with static 12% CP. Previous studies have observed that oscillating CP improved animal production [4] [14] [1]; however, these responses may have been caused by increases in DMI and N recycling via saliva. In the present study we demonstrated that a 48-h oscillation between 10–14% CP was not enough to promote any significant improvement in ruminal N metabolism and microbial efficiency. This suggests that either ruminal microorganisms do not respond to oscillating CP levels or are capable of coping with 48-h periods of undernourishment. It is possible that other levels of CP, other CP sources, or other oscillating regimes could help elucidating these issues.

## Conclusions

Based on our results, oscillating dietary CP between 10–14% at 48-h intervals had no effects on ruminal N metabolism and microbial efficiency when compared with static 12% CP in beef cattle diets. These results indicate that oscillating dietary CP between 10–14% at 48-h intervals do not improve ruminal nutrient digestibility, ruminal fermentation, ruminal N metabolism, and microbial efficiency in beef cattle diets in a dual-flow continuous culture system. This suggests that either ruminal microorganisms do not respond to oscillating CP levels or are capable of coping with 48-h periods of undernourishment. It is possible that other levels of CP, other CP sources, or other oscillating regimes could help elucidating these issues. The diet with 12% CP provided positive effects on microbial N flow and microbial efficiency in the rumen; therefore, it was the best strategy to improve N utilization in the rumen. Beyond that level, there were no further benefits of feeding greater dietary CP.

## Supporting Information

S1 Table Raw Data. (XLSX)

## Abbreviations

ADF: acid detergent fiber
BCVFA: branched-chain volatile fatty acids
CP: crude protein
DM: dry matter
NAN: non-ammonia nitrogen
NH_3_-N: ammonia nitrogen
NDF: neutral detergent fiber
OM: organic matter
OSC: oscillating
RDP: rumen-degraded protein
RUP: rumen-undegraded protein
VFA: volatile fatty acids
